# Effect of manipulating recombination rates on response to selection in livestock breeding programs

**DOI:** 10.1186/s12711-016-0221-1

**Published:** 2016-06-22

**Authors:** Mara Battagin, Gregor Gorjanc, Anne-Michelle Faux, Susan E. Johnston, John M. Hickey

**Affiliations:** The Roslin Institute and Royal (Dick) School of Veterinary Studies, The University of Edinburgh, Easter Bush, Midlothian, Scotland, UK; Institute of Evolutionary Biology, University of Edinburgh, Charlotte Auerbach Road, Edinburgh, EH9 3FL UK

## Abstract

**Background:**

In this work, we performed simulations to explore the potential of manipulating recombination rates to increase response to selection in livestock breeding programs.

**Methods:**

We carried out ten replicates of several scenarios that followed a common overall structure but differed in the average rate of recombination along the genome (expressed as the length of a chromosome in Morgan), the genetic architecture of the trait under selection, and the selection intensity under truncation selection (expressed as the proportion of males selected). Recombination rates were defined by simulating nine different chromosome lengths: 0.10, 0.25, 0.50, 1, 2, 5, 10, 15 and 20 Morgan, respectively. One Morgan was considered to be the typical chromosome length for current livestock species. The genetic architecture was defined by the number of quantitative trait variants (QTV) that affected the trait under selection. Either a large (10,000) or a small (1000 or 500) number of QTV was simulated. Finally, the proportions of males selected under truncation selection as sires for the next generation were equal to 1.2, 2.4, 5, or 10 %.

**Results:**

Increasing recombination rate increased the overall response to selection and decreased the loss of genetic variance. The difference in cumulative response between low and high recombination rates increased over generations. At low recombination rates, cumulative response to selection tended to asymptote sooner and the genetic variance was completely eroded. If the trait under selection was affected by few QTV, differences between low and high recombination rates still existed, but the selection limit was reached at all rates of recombination.

**Conclusions:**

Higher recombination rates can enhance the efficiency of breeding programs to turn genetic variation into response to selection. However, to increase response to selection significantly, the recombination rate would need to be increased 10- or 20-fold. The biological feasibility and consequences of such large increases in recombination rates are unknown.

**Electronic supplementary material:**

The online version of this article (doi:10.1186/s12711-016-0221-1) contains supplementary material, which is available to authorized users.

## Background

In this study, we performed simulations to explore the potential of manipulating recombination rates to increase response to selection in livestock breeding programs. Response to selection in a breeding program is affected by accuracy of selection, generation interval, intensity of selection, and the amount of genetic variation that is available to be selected upon. In recent years, the availability of genomic information has increased the breeders’ ability to manipulate the first three of these factors. Use of genomic information can increase the accuracy of selection by enabling more informative analyses of the data; it can shorten generation interval by allowing accurate assessment of the Mendelian sampling term of selection candidates early in life; and it can increase selection intensity by reducing the cost of evaluating an individual.

Applications of genomic selection have led to increased responses to selection in several breeding programs (e.g., dairy cattle [1] and layer chickens [2]). However, the upper limits of its impact on selection response are likely to be reached in the near future since accuracy asymptote, cost constraints and generation interval cannot be reduced further without adopting new reproduction techniques. These constraints suggest that manipulating the amount of genetic variation that is available for selection will become an important goal for breeders in attempts to further increase response to selection.

The amount of genetic variation that is available to be selected upon in a large random mating population depends on the number of quantitative trait variants (QTV) and their frequencies and effect sizes [3]. When populations are not large and mating is not random, the amount of genetic variance also depends on the degree of linkage between QTV, which limits the frequency of particular combinations of alleles in a population. This is especially true in populations that have undergone directional selection, which induces negative disequilibrium between QTV, i.e., the Bulmer effect [4]. For example, in the simulation study of Gorjanc et al. [5], the Bulmer effect was estimated by subtracting the additive genetic variance (variance of breeding values) from the additive genic variance (variance of breeding values assuming that QTV are completely unlinked). These authors reported additive genic and genetic variances of 0.28 in an unselected base population and 0.22 and 0.16, respectively, after ten generations of random mating and ten generations of selection. If all QTV segregated independently and/or there would be no selection, the genetic variance would be greater. However, QTV do not segregate independently because meiotic recombinations along a chromosome are rare events. Recombination breaks down physical linkages between loci on a chromosome and, on average, only one such event occurs on a typical chromosome; chromosomes of domesticated livestock species are typically one Morgan long, e.g. the average chromosome length in cattle is 0.97 Morgan [6, 7], 1.1 Morgan in pigs [8], 0.91 Morgan in chicken [9], and 1.3 Morgan in sheep [10]. Consequently, the generation of new combinations of alleles is constrained by their arrangement on the chromosomes in any given generation and, thus, the QTV cannot be selected upon in an independent manner.

The idea that recombination provides variation for selection to act upon was first advocated by Weismann in 1889 [11, 12] who proposed that recombination increases the variance of fitness, which after selection leads to increased fitness of the population [13]. Empirical results from several long-term experiments in natural and model organisms have demonstrated that (1) higher rates of recombination in a population result in greater response to selection [12–18]; (2) increased recombination rates evolve as a correlated response to selection when directional selection is placed on some other trait [18–22]; and (3) asexually propagated species have a higher rate of extinction than sexually reproducing species [13]. In addition, it has been shown that domesticated plant species have higher rates of recombination than their ancestors [23], domesticated mammals have higher chiasma frequencies than wild mammals [24, 25], and domesticated pigs have higher recombination rates than wild pigs [26].

Recombination rates vary largely across various scales, i.e. within and between chromosomes [27], individuals [28], species [29], genders [30, 31], and with maternal age [28], and this variation is under both genetic [25] and environmental [32] control. Heritability estimates of recombination rate were found to equal 0.15 in sheep [33], to range from 0.22 to 0.26 in cattle [6, 7], and to be about 0.30 in humans [28, 34]. QTV that affect recombination rate have been detected [35, 36], including one rare variant in humans which increases the average recombination rate by over 10 % in females [34]. Many molecular mechanisms that underlie recombination and the genes involved (e.g., *PRDM9* and *RNF212*) have been discovered and reviewed in, e.g., [37].

Because recombination is under genetic and environmental control, it should be possible to manipulate it, e.g. by including it in total merit selection indices [38], promoting favorable alleles via genome editing [39, 40], carrying out environmental modifications, or perhaps by manipulating it directly. Such manipulations could be used in livestock breeding programs to release more genetic variation in each generation and thus enhance short- and long-term responses to selection.

The aim of this study was (1) to show that higher recombination rates can enhance the efficiency of breeding programs to turn genetic variation into response to selection in the short, medium, and long term, and (2) to determine the recombination rates that would be needed to achieve large increases in response to selection.

## Methods

**Fig. 1:**
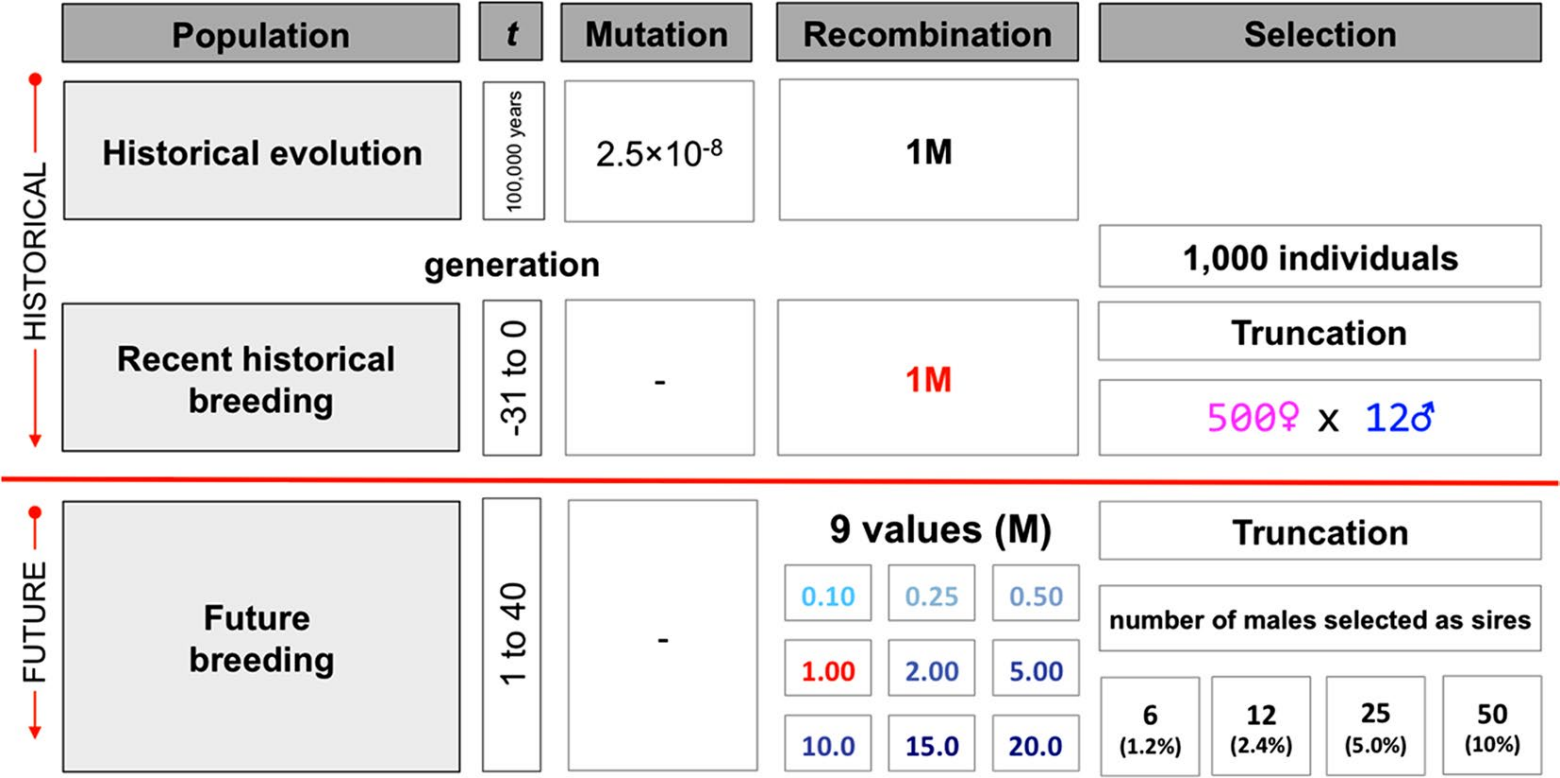
Simulation strategy for manipulation of recombination rates

Simulations were used to evaluate the impact of manipulating recombination rate on response to selection for quantitative traits in livestock breeding programs. Ten replicates of various scenarios were performed. Scenarios followed a common overall structure (Fig. 1) but differed in the average rate of recombination along the genome (expressed as chromosome length in Morgan), the genetic architecture of the trait under selection, and the selection intensity under truncation selection (expressed as the proportion of males selected).

Conceptually, the simulation scheme was divided into historical and future phases. The historical phase represented historical evolution and recent historical animal breeding efforts up to the present day, under the assumption that livestock populations have evolved for tens of thousands of years, followed by 32 recent generations of modern animal breeding with selection on estimated breeding values. The future phase represented 40 future generations of modern animal breeding, in which the breeder was able to select on estimated breeding values and manipulate recombination rates. The historical animal breeding generations were denoted −31 to 0 and the future animal breeding generations were denoted 1–40.

Simulations involved the five following steps:

1. Generating whole-genome sequence data,
2. Generating QTV affecting phenotypes,
3. Generating pedigree structures for a typical livestock population,
4. Performing selection, and
5. Testing the effect of manipulated recombination rates on response to selection.

Results are presented as the mean of the ten replicates for each scenario and encompass response to selection, genetic variance ($\sigma_{A}^{2}$), and genic variance ($\sigma_{\alpha }^{2}$).

### Whole-genome sequence data and historical evolution

Sequence data were generated using the Markovian Coalescent Simulator (MaCS) [41] and AlphaSim [42] for 400 base haplotypes for each of 10 chromosomes in the genome. Chromosomes (each 100 cM long and comprising 10^8^ base pairs) were simulated using a per site mutation rate of 2.5 × 10^−8^, a per site recombination rate of 1.0 × 10^−8^, and an effective population size (N_e_) that varied over time in accordance with estimates for the Holstein cattle population [43] as follows: N_e_ was set to 100 in the final generation of the coalescent simulation, to N_e_ = 1256, 1000 years ago, to N_e_ = 4350, 10,000 years ago, and to N_e_ = 43,500, 100,000 years ago, with linear changes in between these time-points. The resulting sequences had approximately 540,000 segregating sites.

### Quantitative trait variants

A quantitative trait was simulated by randomly sampling 10,000 QTV from the segregating sequence sites in the base population, with the restriction that 1000 QTV were sampled from each of the ten chromosomes. For these QTV, the allele substitution effect was randomly sampled from a normal distribution with a mean of 0 and standard deviation of 0.01 (1.0 divided by the square root of the number of QTV). The effects of the QTV were in turn used to compute true breeding values (TBV) for a trait. We also simulated two other genetic architectures of the trait under selection, using smaller numbers of QTV, either 1000 or 500.

### Pedigree structure, gamete inheritance, and recombination rates

After generating whole-genome sequence data and QTV, a pedigree of 72 generations was simulated. Each generation included 1000 individuals and a portion of these were chosen to be the parents of the next generation by truncation selection. In the first generation of the recent historical animal breeding population (i.e. generation −31), the chromosomes of each individual were sampled from the 400 base haplotypes. In later generations (i.e., generations −30 to 40), the chromosomes of each individual were sampled from parental chromosomes with possible recombination events. Different recombination rates were used, depending on the scenario or generation considered. In all scenarios, the 32 generations of the recent historical animal breeding population (i.e. generations −31 to 0) had a recombination rate of 1 Morgan per chromosome, resulting in a 10-Morgan genome.

In the 40 generations of future animal breeding (i.e. generations 1–40), nine different recombination rates were simulated to create nine scenarios: CL0.10M, CL0.25M, CL0.5M, CL1M_T_, CL2M, CL5M, CL10M, CL15M, and CL20M, where CL refers to the chromosome length, M to the units (Morgan) and T denotes the typical chromosome length in current livestock populations of 1 Morgan. With 10 chromosomes, these scenarios resulted in genome lengths of 1, 2.5, 5, 10, 20, 50, 100, 150, and 200 Morgan. Crossovers were simulated to occur without interference.

### Population history and selection strategies

In the recent historical animal breeding generations (i.e., generations −31 to 0), all 500 females and 2.4 % of the males (i.e. 12 individuals) were selected using truncation selection on their TBV to become the parents of the next generation. In the future animal breeding generations (i.e. generations 1–40), all females were selected and 1.2, 2.4, 5, or 10 % of the males were selected based on their TBV. Different selection intensities were used to change the loss of genetic variance due to selection.

#### Response to selection and variances

Response to selection was calculated in units of the standard deviation of TBV in the base generation ($\sigma_{{TBV_{base} }}$) as $\left( {\overline{{TBV_{curr} }} - \overline{{TBV_{base} }} } \right)/\sigma_{{TBV_{base} }}$, where $\overline{{TBV_{curr} }}$ and $\overline{{TBV_{base} }}$ are the mean TBV in the current and base generation, respectively. Generation 0 was used as the base generation in order to observe the genetic improvement since the start of the future generations of animal breeding.

The genetic variance in each generation was calculated as: $\sigma_{A}^{2} = {\mathbf{a}}^{{\prime }} {\mathbf{a}}/\left( {n - 1} \right)$, where **a** is a zero mean vector of TBV of the *n* individuals in that generation. The genic variance in each generation was calculated as: $\sigma_{\alpha }^{2} = 2\sum\nolimits_{i = 1}^{{n_{QTV} }} {p_{i} q_{i} \alpha_{i}^{2} }$ [3], where *n*_*QTV*_ is the number of QTV, *p*_*i*_ and *q*_*i*_ are the allele frequencies at the *i*-th QTV in a given generation and *α*_*i*_ is the allele substitution effect at the *i*-th QTV. The genetic and genic variances in each generation were expressed relative to the genetic variance in the base generation (thus $\sigma_{A}^{2} = 1$ and $\sigma_{\alpha }^{2} \ge 1$ at generation 0).

#### Design of the specific scenarios

Two different scenarios were constructed to examine specific components of the research objectives.

*Scenario A1* The objective of scenario A1 was to evaluate the impact of a range of recombination rates on response to selection and on reductions in genetic and genic variances with truncation selection across a range of selection intensities and number of QTV controlling the trait under selection. This resulted in a grid of 36 × 3 sub-scenarios (9 recombination rates × 4 selection intensities × 3 traits influenced by different numbers of QTV). The trait influenced by the largest number of QTV was used as a baseline that was compared to traits with other numbers of QTV in some analyses.

**Fig. 2:**
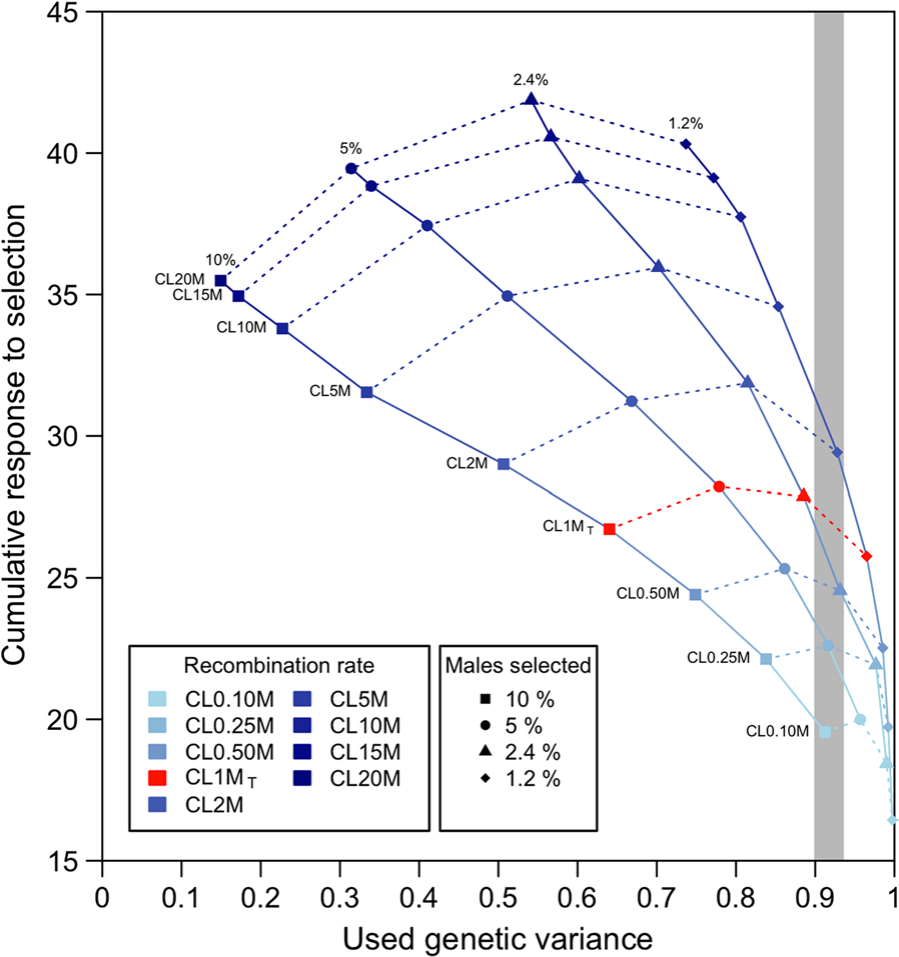
Cumulative response to selection plotted against the used genetic variance at generation 40 for a grid of 36 sub-scenarios with nine recombination rates and four selection intensities for a trait based on 10,000 quantitative trait variants (QTV). Recombination rates are connected with a *solid line*. *Red color* defines the typical chromosome length (CL) of 1 Morgan (CL1M_T_), and the scale of *blue* ranges from low recombination rate (*light blue*, CL0.1M0) to high recombination rate (*dark blue*, CL20M). The different proportions of males selected as sires at each recombination rate are connected by a* dotted line*, and range from 10 % (*square symbol*) to 1.2 % (*diamond symbol*). The *grey area* highlights a set of sub-scenarios that used similar amounts of genetic variance, but produced different levels of response to selection. This set of sub-scenarios was chosen and explored in Fig. 5

*Scenario A2* The objective of scenario A2 was to quantify the additional response to selection that higher recombination rates could provide for the same loss in genetic variance. In scenario A2, the grid of 36 selection intensities and recombination rates from scenario A1 was searched to find sub-scenarios that used genetic variance at similar rates in generation 40, and their response to selection was quantified. These sub-scenarios were identified by visual inspection of Fig. 2 and four of these that are highlighted by the shaded area in Fig. 2, are described in the Results section.

## Results

### Scenario A1: impact of recombination rates

Increasing the recombination rate increased response to selection and decreased the loss of genetic and genic variances. The differences in response to selection and genetic and genic variances between low and high recombination rates increased over generations. At a low recombination rate, cumulative response to selection tended to asymptote sooner and the genetic variance was completely eroded. If the trait under selection was affected by a few QTV, differences in response to selection between low and high recombination rates still existed, but the selection limit was reached for all rates of recombination.

Figure 2 summarizes cumulative responses to selection plotted against the amount of genetic variance that was used up to the last generation of selection (generation 40) for the 36 sub-scenarios of scenario A1, with recombination rate and proportion of males selected as parameters. The nine recombination rates for each selection intensity are connected with a solid line. The four selection intensities (expressed as the proportion of males selected) at each recombination rate are connected with a dotted line. Cumulative response to selection ranged from 16.4 to 41.9; the proportion of genetic variance used ranged from 0.149 (i.e. most genetic variance was preserved) to 0.998 (i.e. most genetic variance was used).

Figure 2 shows that for each selection intensity, increasing recombination rate always increased response to selection and decreased the variance used. The effect of increasing recombination rate on response to selection was greater when smaller proportions of males were selected (higher selection intensities), which caused the dotted contours in Fig. 2 that connect different selection intensities at the same recombination rate to rotate anticlockwise as recombination rate increased.

Figure 2 also shows that the effect of selecting a smaller proportion of males for breeding, which is currently easier than increasing recombination rate in a typical breeding program, always increased the genetic variance used but its effect on response to selection depended on both the selection intensity and the recombination rate. These effects can be summarized by two features of Fig. 2. First, all selection contours had peaks: at the lowest selection intensity, increasing selection intensity always increased cumulative response, whereas increasing to the highest selection intensity always reduced the cumulative response to selection. Thus, there was an optimal selection intensity, i.e. selecting between 2.4 and 5 % of males as sires for the population size simulated here. Second, as noted previously, increasing recombination rate increased cumulative response more at higher selection intensities than at lower selection intensities, which caused the optimal selection intensity to be greater at higher recombination rates, i.e. the optimum shifted from 5 % selected at the lowest recombination rate to 2.4 % at the highest recombination rate.

**Fig. 3:**
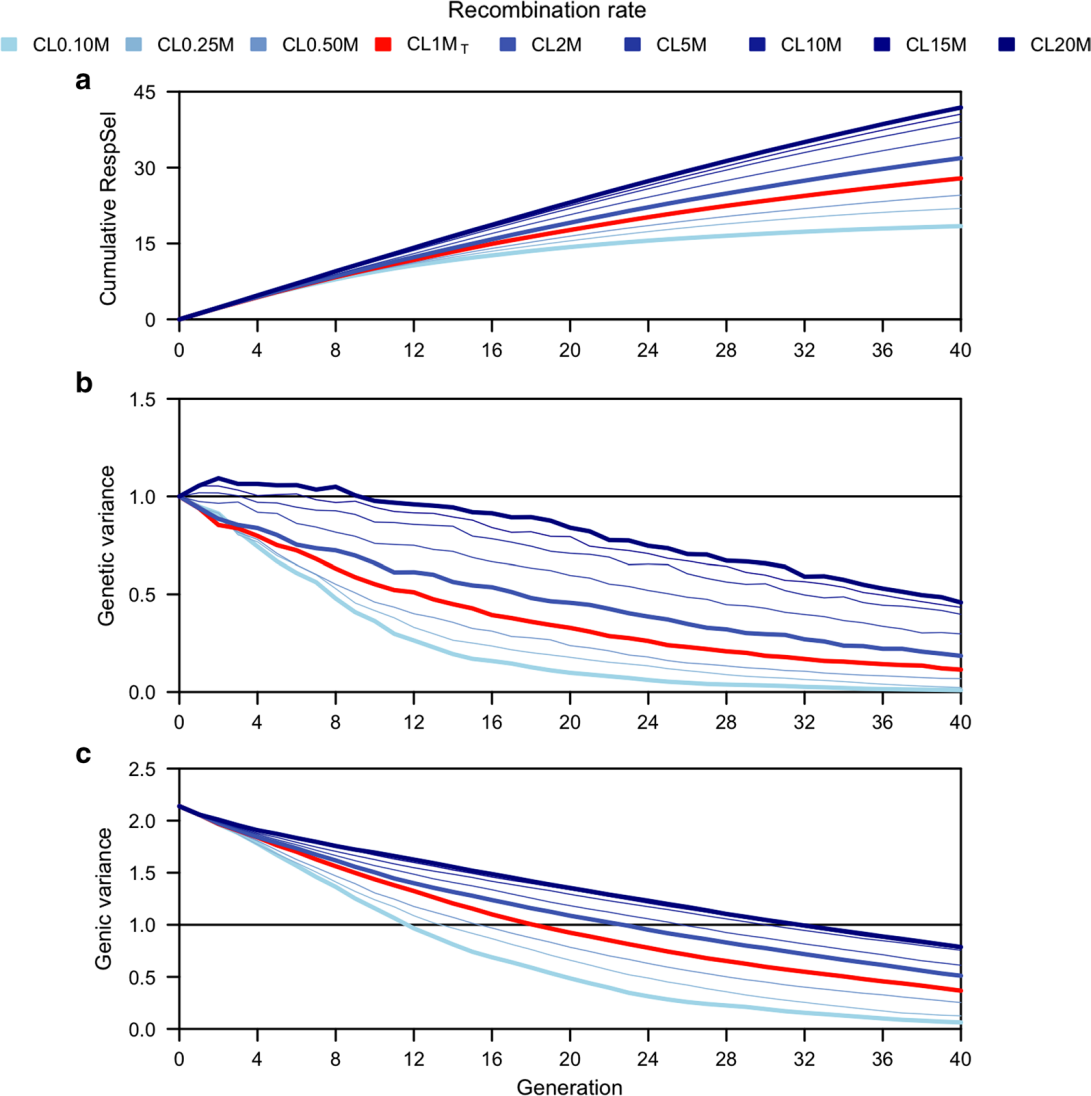
Cumulative response to selection (**a**), genetic (**b**) and genic (**c**) variance for the future breeding populations (from generation 1 to 40).  Cumulative response to selection (**a**), genetic variance (**b**) and genic variance (**c**) for a trait based on 10,000 quantitative trait variants (QTV) are plotted for each generation. From generation −31 to 0 (not plotted), the chromosome length (CL) was equal to 1 Morgan, whereas from generation 1 to 40, the chromosome length ranged from 0.10 Morgan (CL0.10M) to 20 Morgan (CL20M). 2.4 % of the males were selected in each generation by truncation selection. See Additional file 1: Figure S1 for results of all 72 generations

Figure 2 shows cumulative responses and genetic variance used by generation 40 for scenario A1. Figure 3 shows how recombination rate affected cumulative response (Fig. 3a), genetic variance (Fig. 3b) and genic variance (Fig. 3c) during 40 generations of selection, with a fixed selection intensity, for nine recombination rates. Increasing recombination rate increased the positive gradient of cumulative response curves, which shows that recombination enhanced response to selection. Increasing recombination rate reduced the negative gradients of the genetic and genic variance curves, which indicates that less variance was used with high recombination rates.

**Fig. 4:**
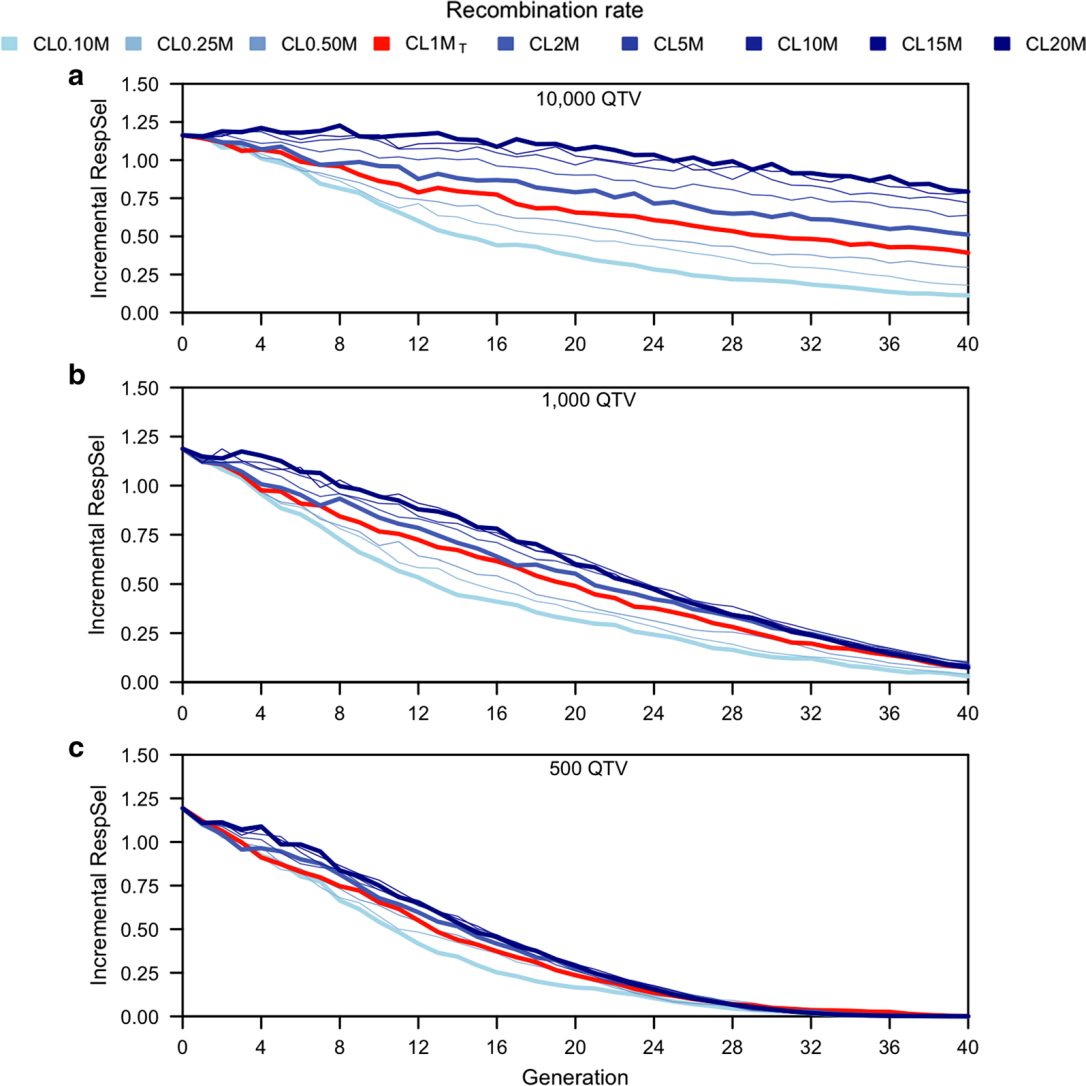
Response to selection by generation for a trait with 10,000 (**a**), 1000 (**b**) and 500 (**c**) quantitative trait variants (QTV). Other details are as in Fig. 3

Very large increases in recombination rate had a large impact on cumulative response to selection, as shown in Fig. 3a. For instance, doubling the chromosome length from the typical 1 Morgan (CL1M_T_) to 2 Morgan (CL2M) increased cumulative response over 40 generations by 12.5 %, while increasing recombination rate 10- and 20-fold increased the cumulative response by 28.7 (CL10M) and 33.4 % (CL20M). At the lowest recombination rate (CL0.10M), cumulative response approached a limit of selection in the long-term; these results are further explored in Fig. 4 (see below).

Genetic variance decreased for all rates of recombination, but more slowly and more variance was preserved at high rates of recombination. At the highest recombination rate (CL20M), genetic variance was still 0.46 in generation 40, whereas at the lowest recombination rate (CL0.10M) it decreased to 0.01 (Fig. 3b). In most cases, the genetic variance decreased monotonically but at the highest recombination rate, it increased in the first few generations: by 9 % in generation 2 for a chromosome length of 20 Morgan but it declined by 9 % in the same generation for a chromosome length of 0.10 Morgan (Fig. 3b).

Genic variance showed roughly the same decreasing trends over time as genetic variance, except that the decrease was steeper initially and it was monotonic for all recombination rates. At generation 0, the genic variance was equal to 2.14, i.e. more than twice as high as the genetic variance (Fig. 3) and at generation 40, it was equal to 0.79 at the highest recombination rate and substantially lower (0.06) at the lowest recombination rate (Fig. 3c).

Figure 3a shows that after many generations of selection, cumulative response to selection tended to increase more slowly, especially at low recombination rates. Next, we explored how this slow-down depended on the genetic architecture of the trait.

Figure 4 shows that the slow-down in response to selection was more rapid when fewer QTV affected the trait. This figure plots the increase in response to selection between subsequent generations for three different trait architectures, 40 generations of selection, with a fixed selection intensity, and for nine different recombination rates using recombination rate as a parameter. Figure 4a has results for the same number of QTV (10,000) as in Fig. 3a but Fig. 4a shows incremental responses rather than cumulative responses. Figure 4b, c show results for two more extreme trait architectures: 1000 QTV for Fig. 4b and 500 QTV for Fig. 4c. When fewer QTV affect the trait, the incremental responses declined more rapidly. High recombination rates delayed these declines in response, but if the trait was affected by few QTV, responses declined to 0 for all recombination rates by generation 40 (Fig. 4c).

If the trait was affected by many QTV, incremental responses remained high throughout the 40 generations at high recombination rates, while responses decreased rapidly at low recombination rates. At the highest recombination rate (CL20M), responses declined by less than 10 % over the first 20 generations (from 1.15 to 1.07 units) and still was almost 70 % of its initial value (0.79 units) by generation 40. At a low recombination rate (CL0.10M), responses declined more rapidly, reaching 0.37 units at generation 20 (32 % of the initial value) and 0.11 units (10 %) at generation 40 (Fig. 4a).

If the trait was affected by only 1000 QTV (Fig. 4b), differences in response between high and low recombination rates were qualitatively similar but the decline in response over generations was more rapid and ultimately eroded the difference between high and low recombination rates. For instance, Fig. 4b (1000 QTV) shows that at the highest recombination rate (CL20M), incremental response decreased to 52 % of its initial value (0.60 units) by generation 20 and to 6 % (0.07 units) by generation 40. At the lowest recombination rate (CL0.10M), incremental response was equal to 0.32 units (28 %) in generation 20 and 0.03 (3 %) in generation 40.

Figure 4c shows that the decline in response to selection was even more rapid and the differences between the highest (CL20M) and lowest (CL0.10M) recombination rates were even smaller when the trait that was affected by only 500 QTV. At generation 20, incremental response decreased to 0.29 units (26 % of the initial value) and to 0.17 units (15 %) for the highest and lowest recombination rates, respectively. At generation 40, incremental responses were equal to 0 for all recombination rates.

### Scenario A2: efficiency of turning genetic variation into response

**Fig. 5:**
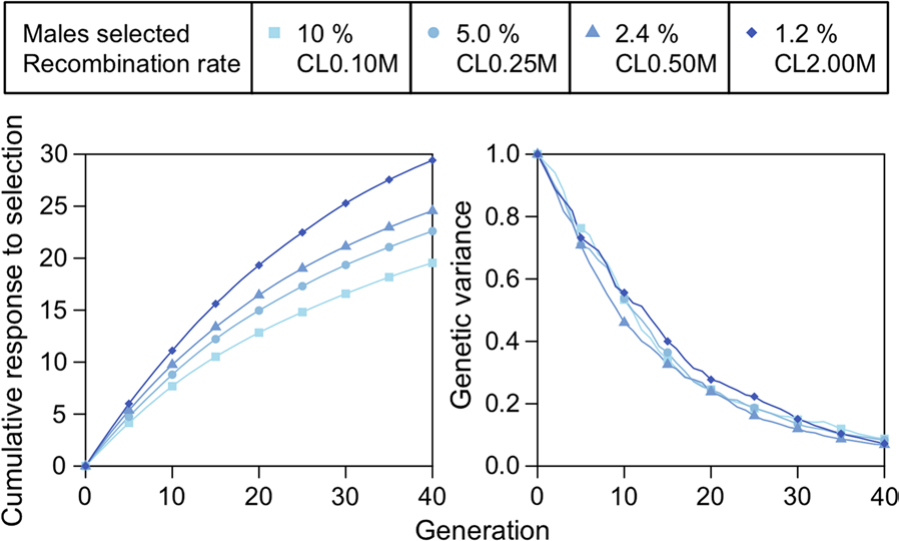
Response to selection and genetic variance for four sub-scenarios of scenario A1 that resulted in the same genetic variance in generation 40 but produced different responses to selection. Other details are as in Figs. 3 and 4

Figure 5 shows that recombination increased the efficiency with which selection turned genetic variation into response by comparing sub-scenarios that produced different responses to selection but used a similar amount of genetic variance; cumulative response is plotted against genetic variance for four such scenarios for 40 generations of selection. These sub-scenarios are marked by the shaded area in Fig. 2. Cumulative responses up to generation 40 for these sub-scenarios ranged from 19.5 to 29.4 and the genetic variance used was almost identical, ranging from 0.91 to 0.93. These sub-scenarios had different recombination rates (chromosome lengths ranged from 0.1 to 2.0 Morgan) and different proportions of males selected as sires (1.2 to 10 %). Scenarios with higher recombination rates and fewer males selected are plotted in darker colors. The scenario with the highest recombination rate (CL2M) was the most efficient at turning genetic variation into response in the short, medium, and long term. For the same genetic variance used, cumulative responses were 1.45, 1.50 and 1.51 greater after 10, 20 and 40 generations than for the scenario with the lowest recombination rate.

## Discussion

The results showed that higher recombination rates can enhance the efficiency of breeding programs at turning genetic variation into response to selection in the short, medium, and long term. Greater response is achieved in the short term because higher recombination rates allow QTV to segregate with a greater degree of independence, which results in more of the genetic variation being accessible to selection in each generation. Greater response is achieved in the long term because fewer favorable alleles are lost from the population as a result of drift. A high recombination rate reduces the effect of drift because the variation among gametes is greater. Greater variation among gametes decreases occurrence of the same permutations of favorable and unfavorable alleles repeatedly among the selected individuals. A high recombination rate will shuffle alleles to a greater degree than a low recombination rate, thus reducing repeated occurrence of the same permutations. Greater variation among gametes also increases the probability that a more representative sample of all alleles is passed on to the next generation. Our study did not allow us to disentangle the effects of these two processes.

Although increased recombination rates were universally associated with greater responses to selection, very large increases in recombination rate were required to generate very large increases in response; doubling the recombination rate resulted in a 12.5 % increase in cumulative response over 40 generations, whereas increasing it 20-fold still only resulted in an 33.4 % increase in response. In addition, differences in responses between recombination rates were marginal in earlier generations and only became more pronounced at later generations, possibly due to reduced loss of genetic variance from the population before it could be selected upon when recombination rates were higher.

Differences in response to selection due to recombination rate raise three main points for discussion i.e. (1) the feasibility of large increases in recombination, (2) accounting for recombination rate in breeding programs, and (3) implications of greater recombination rates for genomic selection.

### Feasibility of large increases in recombination rates

Our simulation results suggest that large increases in recombination rates would be beneficial for increasing selection responses. However, very high recombination rates in domestic populations likely have several important biological downsides and mechanistic limitations. Although recombination rates can be very high in fungi and unicellular organisms (>20 Morgan per chromosome [44]), such high recombination rates are rarely observed in multi-cellular eukaryotes, with the exception of social hymenoptera, such as honeybees (e.g. ~5 Morgan per chromosome [44, 45]). Indeed, recombination rates in mammals appear to be constrained to ~1 crossover per chromosome or chromosome arm [25, 46], with the most extreme values observed in domestic sheep (1.3 crossovers per arm [10]).

The rarity of high recombination rates in mammals may be due to mechanistic trade-offs between the benefits and costs of meiotic recombination. The main benefits are reduced aneuploidy and, possibly, increased fertility in females [28]. The main costs are increased risk of mutation and chromosomal rearrangements, which are associated with disease [25, 47]. From selective breeding and evolutionary perspectives, although recombination may be beneficial for uncoupling deleterious and favorable alleles [48–51], it may also break up favorable combinations of alleles that have been built up by selection [52]. Even so, there is little information on the relative costs and benefits associated with variation in and the magnitude of recombination rates, due to a lack of empirical data that examine associations of recombination rates with offspring viability and other fitness traits.

Our work assumed that recombination occurred without interference. Interference in genetic recombination reduces the occurrence of recombination in nearby chromosomal intervals and the effect of interference decreases as the distance between the chromosomal intervals increases [25, 29]. The assumption of no interference is likely to have caused our simulations to display a greater benefit from increasing recombination compared to what may be observed in practice. We believe that this difference is probably small and affected by unknown, but potentially more important factors, such as the degree to which QTV are clustered in chromosomal regions.

### Accounting for recombination rate in breeding programs

The rate at which genetic variation is used in a livestock breeding population has been an active area of research for several years and several methods have been developed to control it by reducing the co-ancestry between selected individuals [53–55] or by increasing the selection emphasis that is placed on rare alleles [56–58]. Manipulation of recombination rate represents another route through which genetic variation could be maintained and efficiently turned into response to selection. A greater recombination rate facilitates the maintenance of genetic variation in populations under directional selection through increased variation among selection candidates. Maintaining genetic variation in such a population is achieved by breaking negative gametic phase disequilibrium (the “Bulmer-effect”) between the QTV. This greater variance can be used both for short-term goals (there is more variation to select from due to greater variation among gametes and therefore among selection candidates) and for long-term goals (more variation is retained due to inbreeding being localized to regions around QTV). This unlocking of genetic variation was evident in this simulation study. For example, after 30 generations of the conventional breeding program, the additive genetic variance (variance of breeding values) was practically 50 % of the additive genic variance (variance of breeding values when the QTV are completely unlinked), which would have been available to breeders if recombination rate had been unlimited.

Achieving large increases in recombination rate (e.g., more than 2 times higher than currently observed) is likely challenging using conventional approaches. Recent genomic studies have indicated that recombination rate is heritable in domestic mammals (e.g., heritability estimates were 0.15 and 0.22 for genome-wide recombination rates in sheep and cattle, respectively [6, 33]) and therefore has the potential to respond to selection. Previously, we undertook a simulation study [38] in which recombination rate was included as a trait in a multiple trait breeding goal. The results of that study indicated that conventional selection based on genomic breeding values would not lead to sufficient increases in recombination rates to generate increases in response to selection for the other traits in the breeding goal, perhaps because that study assumed that recombination was a quantitative trait controlled by 10,000 QTV.

Recent studies showed that most genetic variance in mammalian recombination rates has a simple genetic architecture, which may allow for targeted genome editing of alleles for increased recombination [39, 40]. Studies in sheep and cattle have shown that the genes *PRDM9*, *RNF212*, and *REC8* are involved in global recombination rate variation [6, 33, 59], with relatively large effects. Nevertheless, fixation of high recombination rate variants at *RNF212* and *REC8* in cattle would only translate into recombination rate increases of 14 and 12 %, respectively [6]. Also, *RNF212* is associated with female recombination rate only in sheep and fixing its favorable allele would translate to an increase of 16 % in this sex alone [33]. An important consideration is that in human studies, variants at *RNF212* are associated with sexually antagonistic variation in human recombination rate [34]. If this is also the case for livestock species, then targeted selection or genome-editing at this locus may only partially translate into genetic gain. However, given that selection intensity is generally higher in males than females, it might be more beneficial to increase recombination rates in males than in females. The results of our study showed that increasing recombination rate was more beneficial when selection intensity was high. Such a strategy would have to take differences in recombination rate between sexes in a given species into account [8].

### Implications of greater recombination rates for genomic selection

Higher rates of recombination will represent a challenge for genomic selection as it is currently implemented because it uses correlations between SNPs and causal variants via linkage and linkage disequilibrium [60] to drive accurate predictions of breeding values. These correlations are reduced by increasing recombination rate, thus leading to lower accuracy of genomic selection, which in turn would reduce the benefit of increasing recombination rate. Therefore, large datasets (i.e. many hundreds of thousands or millions of individuals) with sequence and phenotype data may be needed to maximally benefit from increased recombination rates in breeding programs using genomic selection. Such datasets will ensure that the accuracy of genomic breeding values will depend less on the correlations between SNPs and causal variants because more of the causal variants will be finely mapped. One benefit from greater recombination rate for statistical estimation of allele effects will be lower correlations between the causal variants themselves and between causal variants and nearby neutral variants.

### Implications for quantitative genetic theory

One of the reviewers of our paper pointed out that the observed values of genetic variance and genic variance in the simulations are surprising. Specifically, according to quantitative genetics and selection theory, genetic variance is expected to be smaller than the genic variance [4, 61], which is observed with our results (Fig. 3; Additional file 1: Figure S1). The lower genetic variance is caused by the build up of negative covariances between causal loci brought about by directional selection, i.e., the Bulmer effect [4]. Furthermore, based on the infinitesimal genetic model and ignoring linkage, the genetic variance is expected to be at least half of the genic variance [62], which was not observed in our results (Fig. 3; Additional file 1: Figure S1). For example, after 12, 22, and 32 generations of “historical” breeding, the genetic variance was respectively equal to 65, 52, and 47 % of the genic variance (Fig. 3; Additional file 1: Figure S1). By the end of the simulation (after a total of 72 generations of selection), genetic variance was only 31 % of the genic variance in the baseline scenario (1 Morgan chromosomes). These results suggest that the genetic variance decreased much faster than is expected based on the observed declines in genic variance and the extensively used infinitesimal genetic model without accounting for linkage. However, Bulmer has already shown that linkage increases the amount of negative covariance among loci [62]. Since our work is based on simulated chromosomes with linked loci, it is expected that genetic variance decreases at a faster rate than genic variance. We reran one replicate of the simulation with chromosomes of 1000 Morgan in length and observed a considerably smaller rate of decrease in genetic variance, which is in line with the theory. These observations might have important implications for the often-used breeder’s tools, such as pedigree relationship matrix [63] and selection index (e.g., [64]). These tools largely ignore the effect of linkage and further research is needed to exactly quantify the impact of linkage on them.

## Conclusions

Increasing recombination rates is expected to enhance the efficiency of breeding programs in turning genetic variation into response to selection by using genetic variation more efficiently and reducing the loss of favorable alleles due to selection and drift. However, to obtain large increases in response to selection, recombination rates would need to be increased 10- to 20-fold, and the biological feasibility and consequences of such large increases in recombination rate remain unknown. Traditional selection methods are unlikely to be sufficient for increasing recombination rate to a large degree. Thus, it may be necessary to consider genome-editing approaches to achieve substantial increases in recombination rates.

## Additional file

10.1186/s12711-016-0221-1 Results for the recent historical (from generation −31 to 0) and for the future breeding population (from generation 1 to 40). Mean TBV (a), unscaled genetic variance (b) and unscaled genic variance (c) for a trait based on 10,000 QTV are plotted for each generation. From generation −31 to 0, the chromosome length (CL) was equal to 1 Morgan, whereas from generation 1 to 40 the chromosome length ranged from 0.10 Morgan (CL0.10M) to 20 Morgan (CL20M). 2.4 % of the males were selected in each generation by truncation selection.
