# Supplementary figures and images for: Effect of manipulating recombination rates on response to selection in livestock breeding programs
Source: Genet Sel Evol. 2016 Jun 22;48:44. doi: 10.1186/s12711-016-0221-1 (PMC4917950; doi:10.1186/s12711-016-0221-1)

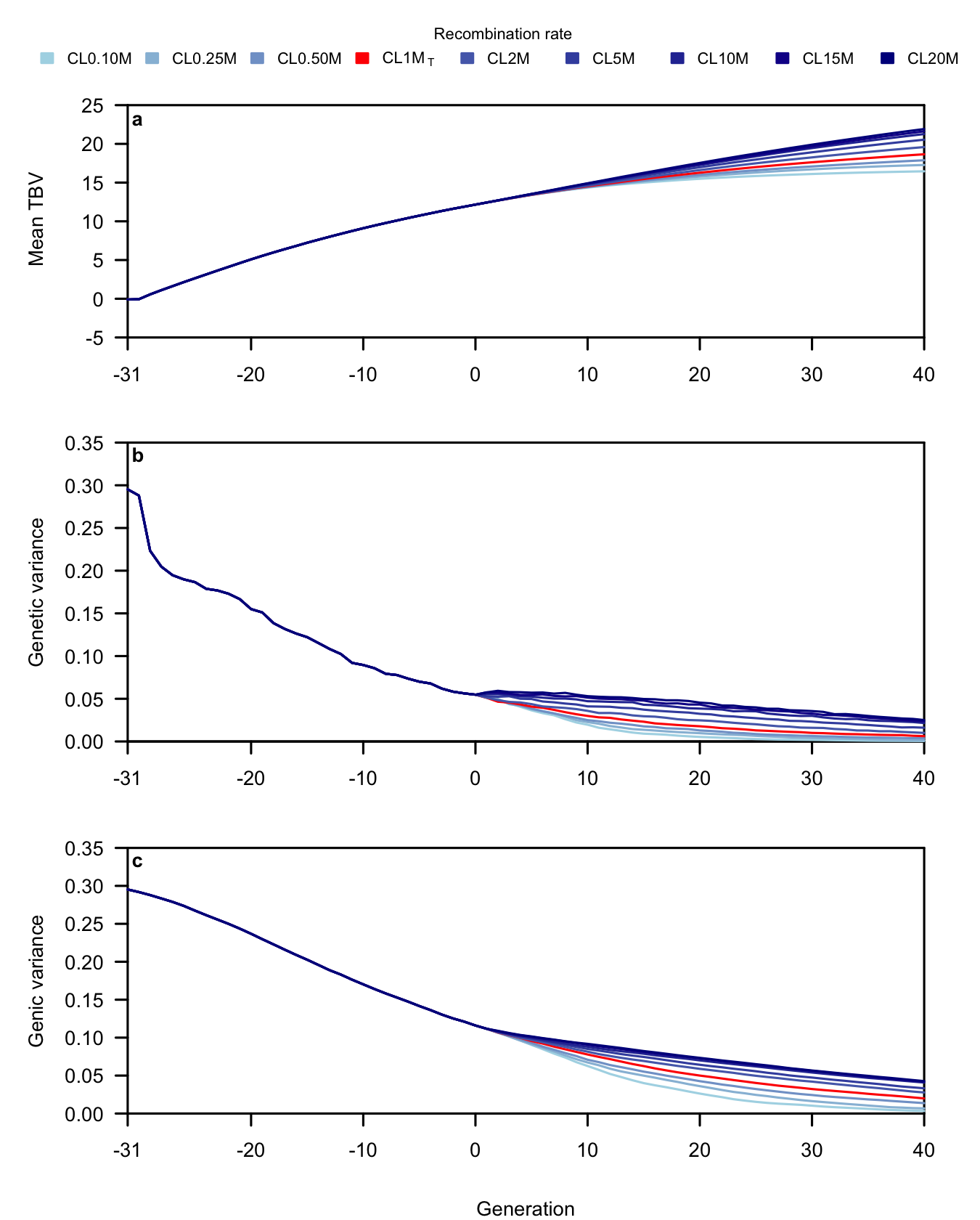

Supplement: Supplementary file 1 — 10.1186/s12711-016-0221-1 Results for the recent historical (from generation −31 to 0) and for the future breeding population (from generation 1 to 40). Mean TBV (a), unscaled genetic variance (b) and unscaled genic variance (c) for a trait based on 10,000 QTV are plotted for each generation. From generation −31 to 0, the chromosome length (CL) was equal to 1 Morgan, whereas from generation 1 to 40 the chromosome length ranged from 0.10 Morgan (CL0.10M) to 20 Morgan (CL20M). 2.4 % of the males were selected in each generation by truncation selection. [file 12711_2016_221_MOESM1_ESM.docx]
